# Phytoestrogens modulate hepcidin expression by Nrf2: Implications for dietary control of iron absorption

**DOI:** 10.1016/j.freeradbiomed.2015.11.001

**Published:** 2015-12

**Authors:** Henry K. Bayele, Sara Balesaria, Surjit K.S. Srai

**Affiliations:** Department of Structural and Molecular Biology, Division of Biosciences, University College London, Gower Street, London WC1E 6BT United Kingdom

**Keywords:** ARE, antioxidant response element, Bach1, BTB and CNC homology 1 basic leucine zipper transcription factor 1, BRG1, Brahma-related gene-1, BRM, Brahma, ChIP, chromatin immunoprecipitation, Ct, cycle threshold, c-Jun, Jun proto-oncogene, c-Fos, Finkel-Biskis-Reilly osteogenic sarcoma, DMSO, dimethyl sulfoxide, EMSA, electrophoretic mobility shifts assay, ER, endoplasmic reticulum, FeNTA, Fe(III) nitrilotriacetic acid, IL-6, interleukin 6, LPS, lipopolysaccharide, MT-1, metallothionein 1, GAPDH, glyceraldehyde 3-phosphate dehydrogenase, GST, glutathione S-transferase, Hamp, hepcidin antimicrobial peptide, HepcARE, Hepcidin antioxidant response element, HO-1, heme oxygenase 1, IgG, immunoglobulin G, Keap1, Kelch-like ECH-associated protein 1, Maf, musculo-aponeurotic fibrosarcoma, QR1, quinone reductase 1, β-TrCP, β-transducin repeat-containing protein, SCF, Skp, Cullin, F-box, Nrf2, NF-E2-related factor 2, PCR, polymerase chain reaction, qPCR, quantitative polymerase chain reaction, tBHQ, tert-butyl hydroquinone, RT-PCR, Real time-PCR, RIPA, Radio-immunoprecipitation assay, Oxidative stress, Iron overload, Hepcidin, Antioxidant response element, Nrf2, Phytoestrogen, Polyphenol, Redox regulation

## Abstract

Hepcidin is a liver-derived antimicrobial peptide that regulates iron absorption and is also an integral part of the acute phase response. In a previous report, we found evidence that this peptide could also be induced by toxic heavy metals and xenobiotics, thus broadening its teleological role as a defensin. However it remained unclear how its sensing of disparate biotic and abiotic stressors might be integrated at the transcriptional level. We hypothesized that its function in cytoprotection may be regulated by NFE2-related factor 2 (Nrf2), the master transcriptional controller of cellular stress defenses. In this report, we show that hepcidin regulation is inextricably linked to the acute stress response through Nrf2 signaling. Nrf2 regulates hepcidin expression from a prototypical antioxidant response element in its promoter, and by synergizing with other basic leucine-zipper transcription factors. We also show that polyphenolic small molecules or phytoestrogens commonly found in fruits and vegetables including the red wine constituent resveratrol can induce hepcidin expression *in vitro* and post-prandially, with concomitant reductions in circulating iron levels and transferrin saturation by one such polyphenol quercetin. Furthermore, these molecules derepress hepcidin promoter activity when its transcription by Nrf2 is repressed by Keap1. Taken together, the data show that hepcidin is a prototypical antioxidant response or cytoprotective gene within the Nrf2 transcriptional circuitry. The ability of phytoestrogens to modulate hepcidin expression *in vivo* suggests a novel mechanism by which diet may impact iron homeostasis.

## Highlights

• Nrf2 regulates *HAMP* transcription.
• Phytoestrogens differentially induce *HAMP* transcription by Nrf2.
• Post-prandial hepcidin induction reduces serum iron and transferrin saturation.
• Nrf2 knockout results in iron-overload in mouse embryonic fibroblasts.

## Graphical abstract

**Figure:**
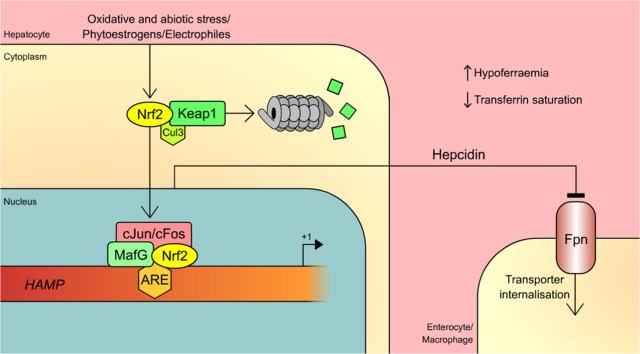

## 1 Introduction

Although hepcidin has almost exclusively been regarded as an iron-regulatory hormone [1], some studies suggest that it may be functionally promiscuous. For example, our recent finding that zinc and cadmium can induce hepcidin expression [2], suggests that it might be a sensor-regulator of (other) heavy metal toxins. Hepcidin induction by these metals indicated parallels with *MT-1*, an Nrf2 target gene that is synergistically up-regulated by metal-responsive transcription factor -1, MTF-1, to confer protection against cadmium toxicity [3], [4]. In addition, fish in habitats that are highly polluted with industrial chemicals expressed more hepcidin than their fresh-water counterparts [5]. Recent reports also suggest that ER stress regulates hepcidin expression [6], [7], an indication that it may be a broad-spectrum stress-inducible cytoprotective peptide.

As the master regulator of cellular stress responses, Nrf2 confers protection against xenobiotic toxicity, tissue injury and acute inflammation by regulating the expression of cytoprotection genes. As a heterodimer with the small Maf proteins (e.g. MafG), Nrf2 binds to the cognate electrophile or antioxidant response element to activate these genes. The signature for the ARE is the core sequence TGACnnnGC (where n is any nucleotide); mutations within the conserved TGAC or GC boxes severely attenuate the antioxidant response [8], [9]. Although primarily associated with phase II genes, this element has also been identified in genes that control processes as diverse as innate immunity, iron metabolism, inflammation and wound healing [10], [11], [12]. Resistance to oxidative or xenobiotic stress is severely compromised in Nrf2 knock-out mice because of depleted cytoprotective protein levels [13], [14]. In spite of its importance in organismal and cellular protection, Nrf2 is repressed under normal conditions by Keap1, a Cullin3-dependent ubiquitin ligase adaptor protein that sequesters Nrf2 in the cytoplasm and targets it for ubiquitination and proteasomal degradation [15], [16]. In addition to Keap1, caveolin-1, SCF/β-TrCP and RXRα also interact with and repress Nrf2 [17], [18], [19].

We hypothesized that Nrf2 might be a critical node that integrates oxidative stress responses to iron toxicity on one hand [20], [21], and the inflammatory response [1] on the other. We reasoned that hepcidin might be a member of the battery of genes that is involved in coordinating those responses. In this report, we show that Nrf2 may coordinate all the signals for hepcidin expression and function. We further found that phytoestrogens (referred to interchangeably here as polyphenols) modulate hepcidin expression both *in vitro* and *in vivo*. In the latter, this induced changes in systemic iron levels. This has consequences for understanding how diet may affect iron homeostasis.

## 2 Materials and methods

### 2.1 Plasmid constructs

We amplified two overlapping fragments of ~700 bp and ~1.8 kb of the *HAMP* promoter from human genomic DNA with the sense primer CATGGTACCAACATCCCCGGGCTCTGGTGACT. Antisense primers for the 1.8 kb and 700 bp promoter fragments were CATCTCGAGCGAGGAGGAGGAGGAGCA and CATCTCGAGGCACCAACTCAGCCTGTGCTGCC respectively. All primers were derived from GenBank Acc # AD000684. The PCR products were digested with *Kpn*I and *Xho*I (restriction sites are underlined), purified with Geneclean (BIO101) and respectively ligated into pGL3 Basic or pGL3 Promoter vectors (Promega), to generate HepcP1.8luc and HepcP0.7luc.

To generate plasmids with putative *HAMP* antioxidant response elements, we synthesized the following phosphorylated complementary oligonucleotides derived from the gene: sense, CTAGCGAATTCAACATCCCCGGGCTCTG**GTGACTTGGC**TGACACTGC; antisense, TCGAGCAGTGTCA**GCCAAGTCAC**CAGAGCCCGGGGATGTTGAATTCG (*Nhe*I*-Xho*I half-sites are underlined); the ARE core is in bold-face. A unique *Eco*RI site was included for linearization in order to confirm insertion of the duplex oligonucleotide. An oligonucleotide with a mutant *ARE* HepcAREMt, in which the ARE core was mutated to GTaACTTGaCT (mutated residues are in lower case) and its complement, were similarly synthesized. Both pairs of oligonucleotides annealed as previously described [21], and ligated directionally into the *Nhe*I-*Xho*I sites of pGL3 Promoter vector to give HepcARE-luc or HepcAREMt-luc. Both constructs were linearized with *Eco*RI to confirm insertion.

BRG1, BRM and c-Fos, plasmids were obtained from Addgene (Cambridge, MA). Nrf1 and Nrf3 cDNAs were purchased from Thermo Fisher Scientific. All plasmid constructs were sequenced for verification.

### 2.2 Cell culture, transfection and reporter assays

All cell culture media were obtained from Invitrogen. HepG2 cells were obtained from ECACC (Porton Down, UK) and cultured in DMEM (with GlutaMAX-1 and high glucose), 10% FBS and antibiotics and antimycotics. Cell culture, transfections and reporter assays were performed as previously described [22]. For transactivation assays, cells were co-transfected with pcDNA3-Nrf2 and the promoter or enhancer constructs; for trans-repression, pcDNA-Keap1 was co-transfected with pcDNA3-Nrf2 and the promoter constructs. For derepression assays, cells transfected with Nrf2, Keap1 and the promoter plasmids were treated with DMSO or polyphenols. C-Jun and c-Fos were also transfected with HepcP1.8luc alone or with Nrf2 to determine transcriptional synergy in hepcidin regulation.

### 2.3 Animals and treatments

All flavonoids were obtained from Sigma and dissolved in 50% ethanol, 10% DMSO. Male, 9-week old Sprague-Dawley rats (230–290 g) were obtained from the Comparative Biology Unit, UCL, and maintained *ad libidum* on standard RM1 diet (SDS, UK). Animal procedures were in accordance with the British Home Office's Animals (Scientific Procedures) Act, 1986. Rats were injected intra-peritoneally with 1 ml of the flavonoids (50 mg/kg body weight); control animals received 50% ethanol/10% DMSO. After 18 h, the animals were sacrificed; livers were snap-frozen in liquid nitrogen and stored at −80 °C until required. Blood was collected by cardiac puncture into test tubes with or without potassium/EDTA; plasma or serum respectively, were separated from blood cells by centrifugation.

### 2.4 RNA extraction and gene expression analysis

Total liver RNA was extracted with TRIzol (Invitrogen) according to the manufacturer's instructions and 1 *μ*g of each sample was reverse transcribed using the Verso cDNA kit (Thermo Fisher Scientific). RT-PCR was performed using Lightcycler 1.5 (Roche) with GAPDΗ as internal standard. Each reaction was performed in duplicate and contained 10 pmoles of specific primers, 1× SYBR Green Mastermix (Qiagen) and 1 *μ*L of cDNA in a 20 *μ*L reaction. Samples without cDNA were included as negative controls. Rat PCR primer-pairs were as follows: *Hamp*: sense, AGACACCAACTTCCCCATATGC; antisense ACAGAGACCACAGGAGGAATTCTT; *QR*: sense, GCTTTCAGTTTTCGCCTTTG, antisense, GAGGCCCCTAATCTGACC TC; *GST*: sense, AGACATCCACCTGCTGGAAC; antisense, GGCTGCAGGAACTTCTTCAC; *HO-1*: sense, TGCTCGCATGAACACTCTG; antisense, TCCTCTGTCAGCAGTGCC. Quantitative PCR was also performed on HepG2 cells treated with polyphenols. The respective primers were: *HAMP*: sense, CTGCAACCCCAGGACAGAG; antisense, GGAATAAATAAGGAAGGGAGG. *GAPDH*: sense, TGGTATCGTGGAAGGACTC; antisense, AGTAGAGGCAGGGATGATG; *HO-1*: sense, GTTGGCACCATGGAGCGTCCG; antisense, AGCCGTCTCGGGTCACCTGG. In all cases, Ct values were obtained for each gene of interest and the *GAPDH* internal standard. Gene expression was normalised to *GAPDH* and represented as ΔCt values. For each sample the mean of the ΔCt values was calculated. Relative gene expression was normalised to controls with an arbitrary expression level of 1.0.

### 2.5 Recombinant Nrf2 and MafG expression

Nrf2 (subcloned into pGEX5x-1, Amersham) and MafG were expressed as recombinant GST and His-tagged proteins respectively in BL21-CodonPlus (DE3)-RIPL cells (Stratagene). Recombinant MafG was partially purified using the MagneHis Protein Purification kit (Promega) as recommended by the manufacturer. GST-Nrf2 was partially purified with Super-Glu10 resin (Generon). Protein integrity was verified by resolving aliquots on a 4–12% NuPAGE Bis-Tris gel in MES buffer (Invitrogen).

### 2.6 Electrophoretic mobility shift assay

Nuclear extracts were prepared from HepG2 cells treated with DMSO or with various polyphenols for 6 h. Double-stranded HepcARE and HepcAREMt oligonucleotides (described above), were used in mobility shift assays as previously described [21]. To further confirm ARE-binding specificity, mobility shift assays were performed with recombinant MafG and Nrf2 alone or in combination. Nrf2/MafG heterodimers were formed by incubating equal amounts of both proteins in EMSA binding buffer for 30 min at room temperature. Aliquots of the heterodimers were then incubated with labeled ARE probe and resolved as above. Recombinant GST was used as negative control.

### 2.7 Chromatin immunoprecipitation (ChIP) assay

HepG2 cells were treated with DMSO (0.1% final concentration), LPS (1 *µ*g/mL), quercetin (50 *μ*M), sulforaphane (10 *μ*M) and tBHQ (100 *μ*M) for 6 h. Chromatin immunoprecipitation was performed using a ChIP kit as instructed by the manufacturer (Upstate). Chromatin was immunoprecipitated overnight with 10 *μ*g anti-Nrf2 antibody (Santa Cruz); control ChIP sample was incubated with a non-specific IgG (Sigma). DNA was purified using the Geneclean kit (BIO 101). PCR was performed with 2 *μ*L of the eluted DNA using the following primers: AACATCCCCGGGCTCTGGTGACT and GCACCAACTCAGCCTGTGCTGCC. The PCR products were resolved on a 2% 1 X TAE agarose gel.

### 2.8 Western blotting

HepG2 cells were treated for 6 h with DMSO or selected polyphenols, and known hepcidin inducers, namely LPS (10 *µ*g/ml; *E. coli* serovar 0111:B4 Sigma), and 100 ng/mL IL-6 (Peprotech). To determine if iron could also induce Nrf2, cells were treated with various concentrations of FeNTA; cadmium, a known inducer of metal toxicity and of Nrf2 was also included at 10 *µ*M. Total cell lysates were prepared in RIPA buffer (Santa Cruz Biotechnology) supplemented with a protease inhibitor cocktail (Roche). Samples (40 *µ*g each) were electrophoresed on 4–12% NuPAGE Bis-Tris gels, and transferred onto Immobilon-P membranes (Millipore). Nrf2 was detected with an anti-Nrf2 antibody (R&D Systems) and goat anti-mouse IgG HRP-conjugate (R&D Systems) using the Visualizer kit (Upstate Biotechnology) and a Fujifilm LAS-1000 imager (Fuji Film, Tokyo, Japan). Beta actin (internal control) was detected with HRP-conjugated anti-β-actin antibody (Abcam).

### 2.9 Non-haem and serum Iron Measurements

Quantitative measurement of non-haem iron was performed according to the method of Torrance and Bothwell [23]. Results were reported as micrograms iron/gram of tissue dry weight. Serum iron was measured using an iron binding assay kit (Pierce). Results were reported as micrograms of iron/decilitre.

### 2.10 Determination of chelatable iron pool by flow cytometry

Nrf2 wild-type and knockout mouse embryonic fibroblasts (MEFs) were grown on 6-cm dishes (PAA) to confluence in Iscove's modified Dulbecco's medium (Invitrogen) supplemented with 10% FBS, insulin-transferrin-selenium and antibiotics/antimycotics mixtures (Invitrogen). For intracellular iron detection, the cells were washed 3X with PBS and then incubated with PBS (as background fluorescence control) or in PBS with 5 µM and 10 µM Phen Green SK dipotassium salt (Invitrogen) for 20 min. The cells were then washed 3X with PBS and detached with PBS/0.5 mM EDTA for 5 min. After centrifugation for 5 min at 2000 rpm, the cells were resuspended in 1 ml PBS for FACs analysis using a CyAn ADP flow cytometer (Beckman Coulter).

### 2.11 Statistical Analysis

Data were analysed using Microsoft Excel (Microsoft) and graphs were plotted with GraphPad Prism 5 software (GraphPad, San Diego, CA). All data were presented as means of duplicates (±S.E.M).

## 3 Results

### 3.1 Hepcidin transcription by Nrf2 through an antioxidant response element

**Fig. 1:**
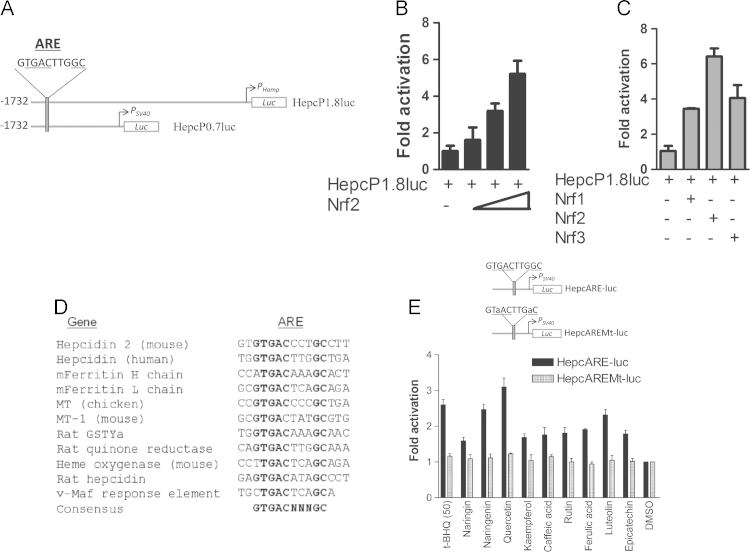
*HAMP* regulation by Nrf2 through an antioxidant response element. **(A)** Promoter constructs. Luciferase expression is driven from the native *HAMP* promoter and transcription start site (*P*_*Hamp*_) in HepcP1.8luc while transcription initiation site in Hepc0.7luc is provided by a minimal SV40 promoter (*P*_*SV40*_). **(B)** Dose-dependent transactivation of *HAMP* promoter by Nrf2. **(C)** Differential activation of *HAMP* promoter by Nrf2 and its isoforms. **(D)** Comparison of HepcARE with the AREs of other cytoprotective genes and putative AREs in hepcidin orthologues: metallothionein, ferritin (mouse, *HO-1, QR1* and *GST-Ya*; the v-Maf recognition sequence is also shown. (**E)** Polyphenols differentially activate HepcARE. HepcARE-luc and its mutant HepcAREMt-luc (both under the control of *P*_*SV40*_) were transfected into HepG2 cells and treated with either DMSO or polyphenols and with tBHQ. Fold-activation is with respect to luciferase expression in DMSO-treated cells.

We hypothesized that hepcidin senses iron because of the oxidative stress that iron generates through Fenton-type reactions. We further conjectured that Nrf2, as the master regulator of stress responses, might regulate hepcidin expression through this mechanism. To examine redox-dependent regulation of hepcidin expression we subcloned two overlapping DNA fragments encompassing ~1.8 kb of the human *HAMP* promoter. We identified a putative hepcidin antioxidant response element (HepcARE) at nucleotides −1732 to −1722 from the initiation codon (Fig. 1**A**). Co-transfections of the promoter into HepG2 cells showed dose-dependent transactivation by Nrf2 (Fig. 1**B**). We also tested Nrf2 isoforms, Nrf1 and Nrf3, for their abilities to transactivate the promoter and found differential activation, with Nrf2 inducing the highest level of promoter activity (Fig. 1**C**). Sequence comparisons showed that HepcARE (see Fig. 1**A**) fits the canonical ARE consensus nTGACnnnGC. This element was also identical to the ARE of rat *QR* and was homologous to the AREs of phase II and other oxidative stress-inducible and cytoprotective genes. Similar sequences and spatial arrangements were also found in the promoters of mouse hepcidin 2 (*mhepc2*) and rat hepcidin genes (Fig. 1**D**). Of the two paralogous mouse hepcidin genes, *mhepc1* is considered functionally equivalent to its human orthologue but is devoid of an ARE in an equivalent position because of a retroviral element inserted within that region of this gene [24]. However a putative ARE is present further upstream of the integration; *mhepc2* has an ARE in a spatial arrangement similar to *HAMP*.

To test whether the putative ARE in *HAMP* was functional, we directionally subcloned it and its mutant into a luciferase vector under the control of the SV40 promoter. Transient transfection of these enhancer constructs showed that the wild-type ARE construct enhanced luciferase expression and that this was differentially inducible by polyphenols compared with DMSO; however this expression was reduced to background expression levels with the mutant ARE construct (Fig. 1**E)**. This confirmed that HepcARE was functional and necessary for responsiveness to prototypical Nrf2 inducers.

### 3.2 Phytoestrogens induce HAMP expression in vitro and in vivo

**Fig. 2:**
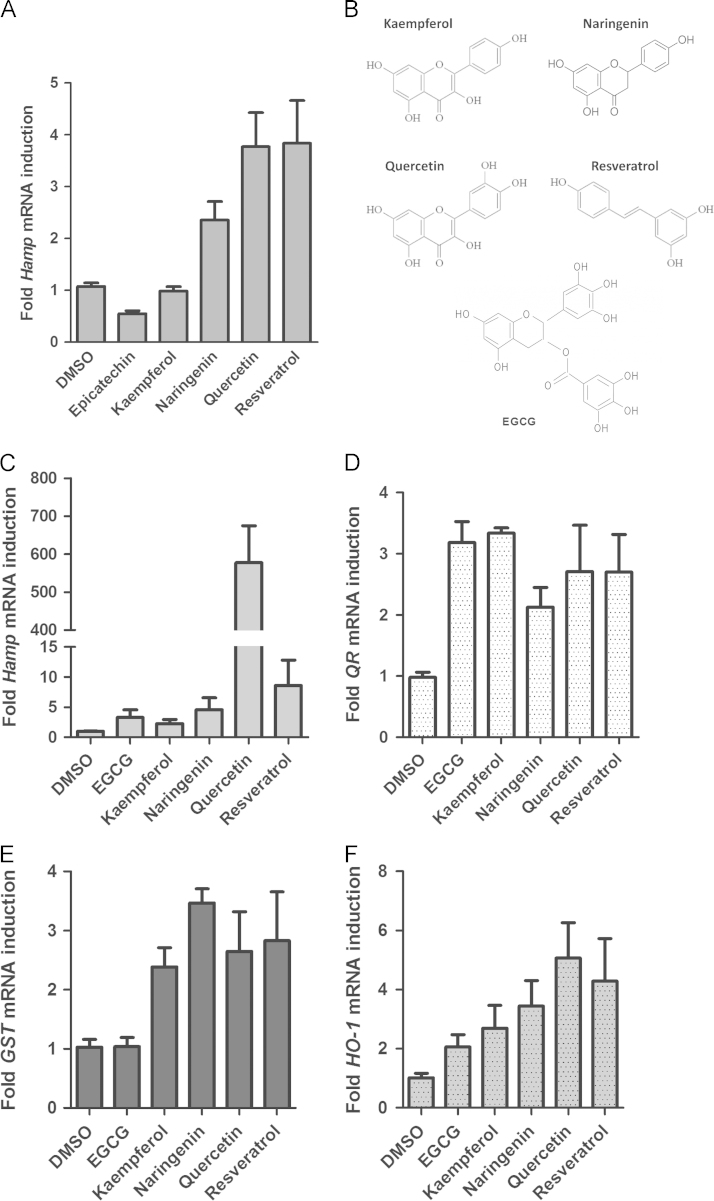
Induction of hepcidin and phase II gene expression by polyphenols. **(A)** RT-PCR of *HAMP* mRNA in HepG2 cells treated with 100 µM polyphenols or DMSO for 6 h. (**B**) Structures of polyphenols used for *in vivo* study. Kaempferol (flavonol), Naringenin (flavanone), quercetin (flavonol), resveratrol (stilbene) and EGCG (tea catechin). Rats were injected intra-peritoneally with either DMSO or with selected polyphenols for 18 h; liver RNA was extracted and analysed by qRT-PCR for the expression of: (**C**) *Hamp*; (**D**) Quinone reductase (*QR1)*; (**E**) Glutathione S-transferase (*GST*), and (**F**) Heme oxygenase 1 (*HO-1)*. All target mRNA levels were normalized to GAPDH internal control.

**Fig. 3:**
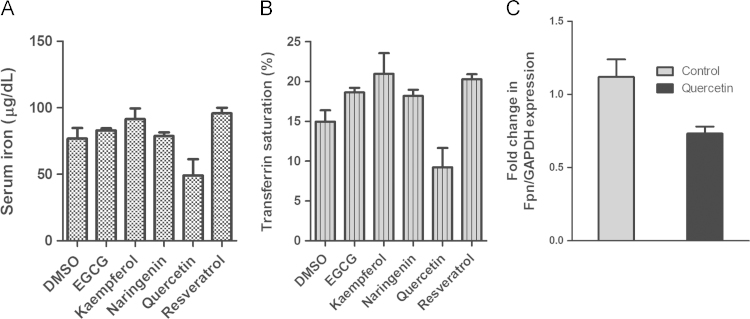
Hepcidin induction by polyphenols *in vivo* correlates with changes in: (A) Systemic iron levels, and (B) Transferrin saturation. (C) Hepcidin up-regulation by quercetin is inversely correlated with *Fpn* expression in rat liver.

Since we found that polyphenolic antioxidants could activate HepcARE, we asked whether they could induce hepcidin expression. To determine this, HepG2 cells were treated with selected phytoestrogens and RNA was extracted after 6 h for RT-PCR. This showed differential induction of *HAMP* mRNA expression by these molecules (Fig. 2**A**); *HO-1* was similarly induced by polyphenols (data not shown). To determine whether phytoestrogens could also induce *Hamp* expression *in vivo*, we injected age-matched rats intra-peritoneally (IP) with a selection of these compounds (Fig. 2**B**): (-)-epigallocatechin-3-gallate (EGCG), kaempferol, naringenin, quercetin and resveratrol; control animals received 0.1% DMSO in normal saline. After 18 h, we performed RT-PCR on liver RNA and found *Hamp* induction in polyphenol-treated rats but not in animals which received only DMSO (Fig. 2**C**). We also found parallel increases in the expression of the phase II genes *GST* and *QR1*, as well as *HO-1* (Fig. 2d**–**F), indicating a common signaling pathway for these genes. While we found only modest induction of all the phase II genes with quercetin, this polyphenol induced a massive increase in hepcidin expression (over 500-fold); this differential effect cannot be easily explained. Although this could be attributed to synergism between Nrf2 and the JAK/STAT3 pathway, we discounted that possibility because quercetin represses IL-6 signaling [25], [26]. We therefore propose that alternative Nrf2 pathways may be involved such as protein kinase pathways [8]. Hepcidin hyper-induction by quercetin correlated with changes in serum iron levels and transferrin saturation in the animals; the other polyphenols did not induce any changes in these parameters (Fig. 3A and B). We also measured hepatic *Fpn* mRNA levels by RT-PCR and found a significant reduction in the livers of quercetin-treated compared with DMSO-fed rats (Fig. 3**C**). Polyphenols induce a large number of microRNAs [27] one of which, miR-17-3p, we previously found to be strongly induced by quercetin [28]. This microRNA represses *Fpn* transcription in intestinal cells by targeting its 3'UTR. We speculate that miR-17-3p rather than hepcidin may have contributed to the post-transcriptional repression of *Fpn* we found. Paradoxically, other work suggests that *Fpn* itself may be up-regulated by Nrf2 in mouse splenic macrophages [29]. However previous reports and our own (unpublished) observations suggest that Fpn regulation by iron or pro-inflammatory stimuli may be cell or tissue-specific, and may not be dependent on hepcidin [30], [31]. The possibility of other iron-regulatory molecules such as lipocalin 2 [32] impinging on the hepcidin-Fpn axis cannot be ruled out.

### 3.3 Derepression of Nrf2 from Keap1 by dietary polyphenols/phytoestrogens

**Fig. 4:**
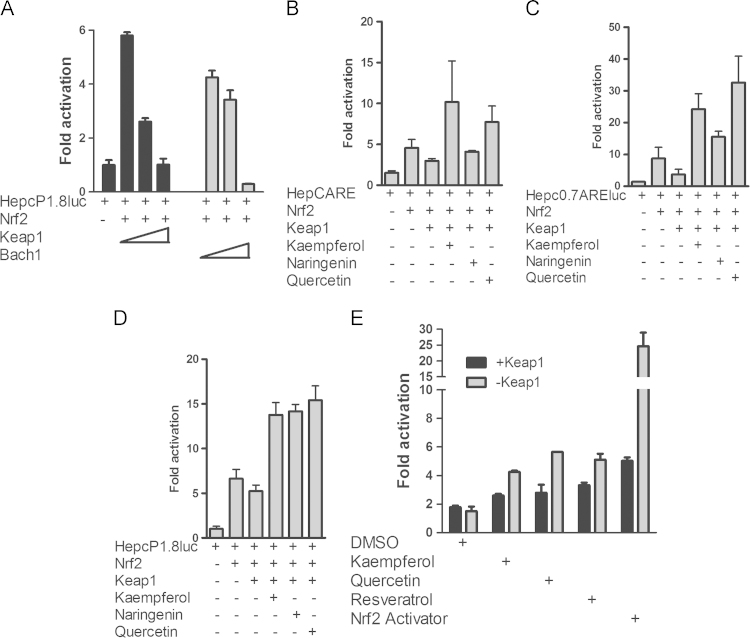
Polyphenols and the Nrf2 activator 2-trifluoromethyl-2′-methoxychalcone derepress Nrf2 from Keap1. (**A**) Keap1 and Bach1 dose-dependently repressed *HAMP* transcription by Nrf2. HepcP1.8-luc was transfected alone or with 100 ng Nrf2 and increasing concentrations of Keap1 or Bach1. (**B**) HepcARE, (**C**) HepcP0.7ARE-luc and (**D**) HepcP1.8-luc were transfected alone or with Nrf2 into HepG2 cells. Where indicated, Keap1 was co-transfected with Nrf2. The cells were treated with DMSO or polyphenols. (**E**). HepcP1.8-luc was co-transfected with Nrf2 and Keap1; cells were treated for 24 h with resveratrol, quercetin and kaempferol or with DMSO and the Nrf2 activator 2-trifluoromethyl-2′-methoxychalcone as negative and positive controls respectively. In all cases except DMSO, Nrf2 was derepressed from Keap1.

As Nrf2 couples with Keap1 to form a redox sensor, we asked if they could regulate hepcidin expression. Co-transfection of Keap1 [14] or Bach1 [33] with the full-length promoter and Nrf2 showed dose-dependent luciferase repression by Keap1 (Fig. 4**A, left panel**). Similarly Bach1 dose-dependently repressed promoter activity (Fig. 4**A, right panel**), consistent with the ability of these proteins to repress Nrf2 target genes. We next asked whether these phytoestrogens could derepress Nrf2 from Keap1. To test this, we transfected cells with the promoter or enhancer constructs and treated the cells with selected phytoestrogens; we found that these compounds differentially relieved Nrf2 repression by Keap1, restoring luciferase expression to levels similar to those with Nrf2 alone (Fig. 4**B–D**). Nrf2 derepression by the phytoestrogens was much higher than could be expected from its simple release from Keap1, suggesting that these compounds may amplify the activity of other components of the Nrf2 signaling pathway. As a positive control, we also tested the chalcone derivative and Nrf2 activator, 2-trifluoromethyl-2′-methoxychalone [34] for *HAMP* promoter derepression from Keap1. This small molecule (at 10 *µ*M) induced promoter activity ~30-fold compared with resveratrol, quercetin, naringenin and kaempferol; Keap1 repressed this effect (Fig. 4**E**).

### 3.4 Nrf2 synergizes with c-Jun to regulate HAMP transcription

**Fig. 5:**
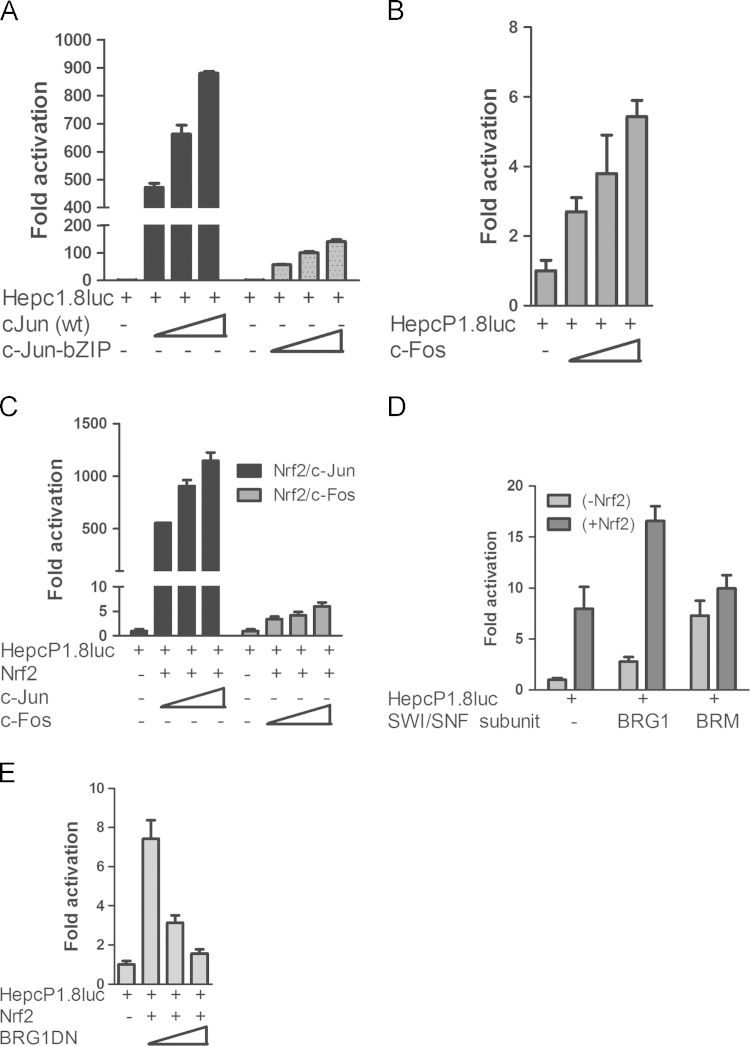
Synergy between Nrf2, bZIP family members and chromatin modifiers in hepcidin regulation. **(A)** Dose-dependent increase in hepcidin transcription by wild-type but c-Jun but not its dominant-negative mutant; **(B)** Comparative hepcidin regulation by c-Fos; **(C)** Transcriptional synergy between Nrf2 and c-Jun but not c-Fos in hepcidin regulation; compare with (A). **(D)** BRG1enhances *Hamp* transcription by Nrf2. Nrf2 was transfected alone or with SW1/SNF subunits BRG1 or BRM. **(E)** Dose-dependent *Hamp* repression by dominant-negative BRG1.

Since Nrf2 interacts with other members of the basic leucine-zipper (bZIP) transcription factor family in redox-dependent gene regulation [35], [36], we asked if c-Jun and/or c-Fos homo- or heterodimers, might co-operate with Nrf2 to regulate hepcidin expression. We transfected the promoter with c-Fos, wild-type c-Jun and its dominant-negative mutant c-JunbZIP; this lacks the dimerization domain. We found that while wild-type c-Jun markedly increased promoter activity, c-JunbZIP was comparatively less potent (Fig. 5**A**); c-Fos activated the promoter less robustly than c-Jun (Fig. 5**B**). To test whether Nrf2 synergizes with c-Jun or c-Fos in *HAMP* transcription, we co-transfected them with HepcP1.8luc. We found that Nrf2-dependent increase in promoter activity was enhanced by c-Jun but not by c-Fos (Fig. 5**C**). Promoter transactivation by Nrf2 and c-Jun was synergistic but non-additive, i.e. promoter activity was higher than a summation of their individual activities.

### 3.5 HAMP transcription by Nrf2 is enhanced by chromatin remodelling factors

Chromatin modifiers of the BAF chromatin-remodelling complex regulate diverse genes including those involved in the inflammatory response [37], [38]. For example, Brahma-related gene 1 (BRG1/SMARCA4), has been shown to regulate *HO-1* transcription [39]. We found that co-transfection of BRG1 and its relative BRAHMA, BRM, increased basal hepcidin promoter activity. However, only BRG1 could enhance hepcidin promoter activity when co-expressed with Nrf2 (Fig. 5**D**) while its dominant-negative mutant BRG1DN repressed it **(**Fig. 5**E)**. Thus although BRM supports higher basal promoter activity than BRG1, compared with the latter it did not synergize with Nrf2 in *HAMP* promoter transactivation. This may be because only BRG1 is capable of interacting with leucine zipper transcription factors (of which c-fos, c-Jun and Nrf2 are members), while BRM prefers ankyrin repeat proteins of the Notch signaling pathway [40], [41]. Thus BRG1 appears to be the preferred partner in Nrf2 transactivation of hepcidin expression.

### 3.6 Nrf2 recruitment to the hepcidin promoter through HepcARE

**Fig. 6:**
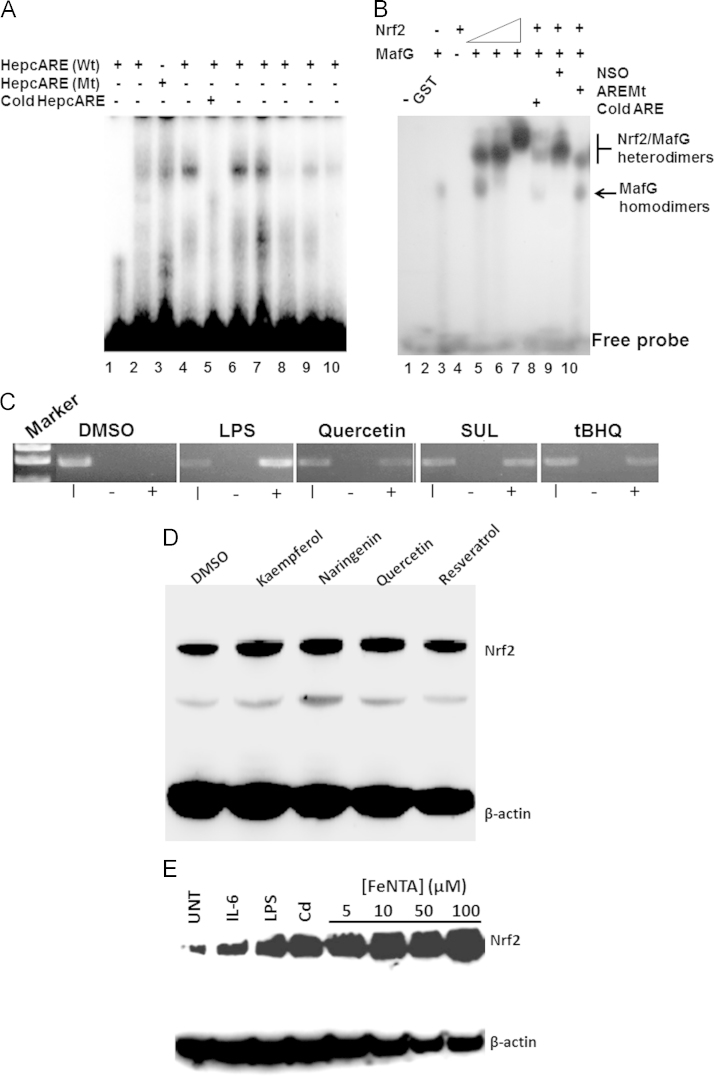
Recruitment of Nrf2 to the hepcidin promoter occurs via the ARE. **(A)** EMSA with HepcARE and nuclear extracts from HepG2 cells treated with 2, DMSO; 4, tBHQ (100 µM); 6, quercetin (50 µM); 7, sulforaphane (10 µM); 8, resveratrol (100 µM); 9, hemin (100 µM) and 10, FeSO_4_ (200 µM). Lane 1 contains HepcARE without nuclear extract; lanes 3 and 5 contained nuclear extract from quercetin-treated cells incubated with HepcAREMt or with 100-fold molar excess of cold HepcARE respectively. **(B)** EMSA with recombinant GST-Nrf2 and His-MafG. 1, HepcARE only; 2, HepcARE plus purified GST (as negative control); 3, HepcARE/His-MafG; 4, HepcARE/GST-Nrf2; 5-7, HepcARE/His-MafG plus increasing concentrations of GST-Nrf2; 8, Competition with excess cold HepcARE interferes with HepcARE binding by His-MafG/GST-Nrf2 heterodimers; 9, Competition with non-specific oligonucleotide (NSO) for GST-Nrf2/His-MafG binding; 10, HepcAREMt plus His-MafG/GST-Nrf2 heterodimers. **(C)** ChIP assay. HepG2 cells were treated with DMSO, 1 μg/mL LPS, quercetin (50 μM), sulforaphane (10 μM), and tBHQ (100 μM). PCR of cross-linked chromatin immunoprecipitated with a non-specific IgG, lane 2 (-), or with an anti-Nrf2 antibody (+), lane 3; lane 1 contains PCR from input chromatin (I). DNA molecular size markers are shown for comparison. (**D)** Nrf2 is induced by polyphenols. Western blot of total lysates of HepG2 cells treated with DMSO or 100 μM kaempferol, naringenin, quercetin, and resveratrol for 6 h. (**E**) Nrf2 is up-regulated by effectors of hepcidin expression. HepG2 cells were treated for 6 h with DMSO or with LPS, IL-6, Cd and with increasing concentrations of FeNTA. β-actin expression was used as internal standard.

To determine the specificity of the interaction between Nrf2 and HepcARE, we performed electrophoretic mobility shift assays using nuclear extracts from HepG2 cells treated with DMSO or selected phytoestrogens, as well as with hemin and Fe^2+^. In these assays we found increased binding of the oligonucleotide using nuclear extracts from polyphenol-treated cells compared with those from DMSO-treated cells (Fig. 6**A**). This binding could be competitively inhibited by excess unlabeled HepcARE (lane 5); mutant HepcARE could not bind to the nuclear component and compared with control we found only modest binding in nuclear extracts from cells treated with FeSO_4_ **(**Fig. 6**A**, lanes 2and 9 respectively). To confirm Nrf2 binding to HepcARE, we used recombinant Nrf2 and MafG in mobility shift assays. While Nrf2 was incapable of binding to HepcARE on its own, MafG homodimers bound to this element. When Nrf2 was incubated at increasing concentrations with MafG, we found a dose-dependent increase in HepcARE binding. This was diminished by competition with cold HepcARE (Fig. 6**B,** lane 8) but not with a non-specific oligonucleotide (lane 9), while the mutant ARE showed reduced binding to Nrf2/MafG heterodimers (Fig. 6**B,** lane 10). This showed conclusively that Nrf2/MafG heterodimers bind to HepcARE.

As a final test of Nrf2 recruitment to the *HAMP* promoter at the genomic level, we performed ChIP assays. Upon treating HepG2 cells with quercetin, and tBHQ and the isothiocyanate sulforaphane, we immunoprecipitated that region of the promoter with HepcARE using an anti-Nrf2 antibody but not from cells treated with DMSO or when chromatin was immunoprecipitated with a non-specific IgG of the same isotype. Interestingly, LPS treatment increased hepcidin promoter occupancy by Nrf2 far more than the phytoestrogens did (Fig. 6**C**); this is consistent with other observations that LPS rapidly induces Nrf2, HO-1 and quinone reductase expression [42].

### 3.7 Hepcidin effectors induce Nrf2 expression

We further confirmed Nrf2 induction by Western blotting; this showed that compared with DMSO, phytoestrogens increased Nrf2 protein levels in HepG2 cells (Fig. 6**D**). We also tested other effectors of hepcidin expression that includes both biotic (IL-6 and LPS) and abiotic (Fe^2+^ and Cd)) inducers to see if they could also induce Nrf2 expression. We treated HepG2 cells with these stimuli, and increasing concentrations of FeNTA. Western blotting showed that compared with untreated controls, Nrf2 was highly expressed in cells treated with IL-6, LPS, and FeNTA as well as by Cd (Fig. 6**E**). Taken together, these observations show that the effectors of hepcidin expression also activate Nrf2; this is consistent with a common signaling pathway.

### 3.8 Nrf2 deletion causes intracellular iron accumulation

**Fig. 7:**
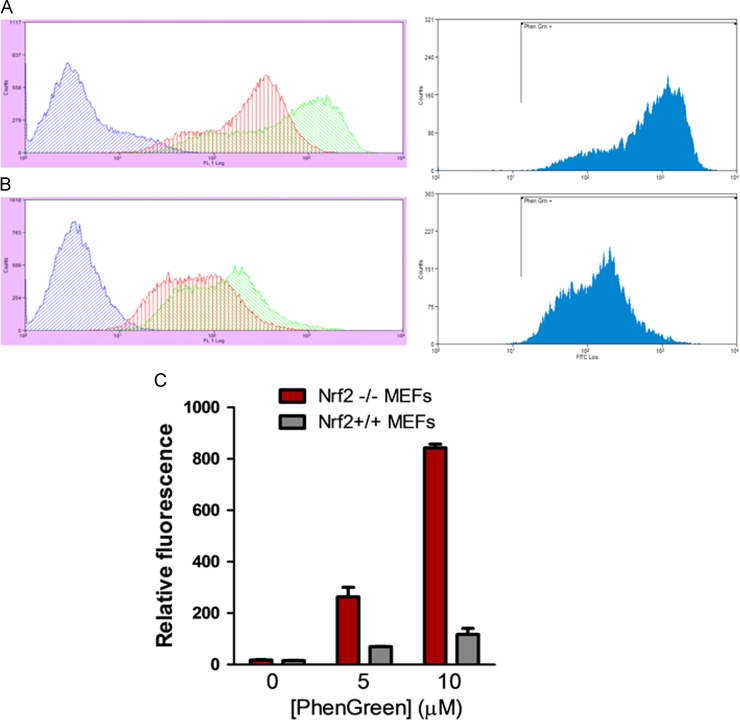
Intracellular staining of labile iron pool shows that Nrf2 regulates iron loading. **(A)** Nrf2−/− (knockout) and (**B**) wild-type (Nrf2 +/+) MEFs were either untreated (blue trace) or treated with 5 µM (red trace) and 10 µM (green trace) Phen Green SK. Cells were subjected to FACs analysis as described above. (**C**) Quantification of relative Phen Green fluorescence (as a measure of the amount of chelatable or labile iron pool) in knockout and wild-type MEFs.

We hypothesized that Nrf2 might regulate iron-overload induced toxicity. To test this we treated mouse embryonic fibroblasts (MEFs) from wild-type and Nrf2 knockout mice with Phen Green SK [43], a green fluorescent indicator of intracellular iron, to determine differences in chelatable iron pools. FACs analysis (Fig. 7**A and B)** showed that Nrf2 knockout MEFs accumulated ~8 times more chelatable labile iron compared with wild-type MEFs (Fig. 7**C)**.

## 4 Discussion

We know much more about the role of hepcidin in iron homeostasis than we do about its designed function as a defensin. Here our observations suggest that Nrf2 might integrate both functions through its role as the primary transcriptional regulator of cellular defense responses. We showed that Nrf2 up-regulated hepcidin transcription from an ARE that bound Nrf2/MafG heterodimers. We also found that polyphenols or phytoestrogens commonly found in fruits and vegetables could induce the expression of hepcidin as well as other defense genes in rats. *In vitro,* these small molecules seemed able to relieve Nrf2 repression as shown by their ability to reactivate hepcidin promoter silencing by Keap1. This may be related to the ability of similar molecules to inhibit Keap1-dependent Nrf2 ubiquitination and proteasomal degradation [44].

While the link between iron and Nrf2 is less obvious than it is with hepcidin, it might be inferred from the ability of free iron to induce oxidative stress. Iron overload causes several diseases for this reason and in all of them, Nrf2 is implicated; these include type 2 diabetes, steatohepatitis, the metabolic syndrome, ageing, inflammatory and cardiovascular diseases, neurodegeneration and cancer [20], [45], [46], [47]. Nrf2 involvement in iron-related diseases is supported by other studies which showed that FeNTA induced nephrotoxicity in Nrf2-knockout but not in wild-type mice. Protection from kidney damage was coincident with the induction of Nrf2 and its target genes, while priming with Nrf2 inducers before FeNTA administration also conferred protection [48], [49]. While this manuscript was in preparation, Silva-Gomes et al. [50] reported that Nrf2 could protect mice against iron-overload induced injury. This supports our findings that MEFs from Nrf2-knockout mice were more susceptible to iron-overload than wild-type MEFs. Further, Nrf2 knockout mice accumulated significantly more liver iron than wild-type mice [51]. Taken together, these findings implicate Nrf2 in iron homeostasis and in preventing iron-related toxicity.

Our observation that LPS increased Nrf2 recruitment to the *HAMP* promoter is consistent with its role in innate immunity, tissue repair and regeneration [11], [12], [52]. Together with other findings of hepcidin induction by ER stress [5], [6], it appears that synergies between Nrf2 and other bZIP proteins such as c-Jun and MafG (as shown in this report) may underlie hepcidin regulation by biotic and abiotic acute stressors. Supporting evidence of hepcidin regulation by the Nrf2-Keap1 axis also come from microarray analyses of mouse liver RNA. Along with other proteins required for cell survival and proteostasis, hepcidin was induced when mice were fed the chemopreventive agent *3H*-1,2-dithiole-3-thione [53]. Our findings therefore suggest that hepcidin regulation may be under the same Nrf2 transcriptional circuitry that guides the expression of first responders for oxidative stress defense and survival; these include detoxification enzymes, ferritin, HO-1 and MT-1 [3], [8], [10], [42], and proteins encoded by the immediate early response genes *c-fos, c-jun* and *c-myc* [54]. Synergistically, these proteins ensure redox balance and protection against oxidative stress-related diseases.

Taken together, this report shows that dietary phytoestrogens may combinatorially control systemic iron levels by up-regulating hepcidin expression, and could (by extension) prevent some of the diseases associated with iron-induced toxicity indicated above. In general, phytoestrogens have a hormetic effect on human health, conferring protection against many oxidative-stress associated pathologies. Some reports suggest that quercetin and related polyphenols such as EGCG may reduce iron toxicity by chelation or by preventing its release/efflux from cells [55], [56]. It was particularly intriguing that of all the compounds tested, quercetin had the most dramatic effect on post-prandial hepcidin induction, serum iron and transferrin saturation. This may partly explain their ability to reduce iron overload in haemochromatosis [57]. Our findings therefore indicate that by inducing hepcidin expression, phytoestrogens may be useful adjunctive nutraceuticals for controlling diseases of iron overload and also for preventing the sequelae of iron-induced toxicity such as hepatitis, cirrhosis, nephrotoxicity and carcinogenesis [44], [47], [48], [49], [58], [59], [60].

## Potential conflict of interest

Nothing to report.

## References

[1] Ganz T. (2003). Hepcidin, a key regulator of iron metabolism and mediator of anemia of inflammation. Blood.

[2] Balesaria S., Ramesh B., McArdle H., Bayele H.K., Srai S.K. (2010). Divalent metal-dependent regulation of hepcidin expression by MTF-1. FEBS Lett..

[3] Klaassen C.D., Liu J., Choudhuri S. (1999). Metallothionein: an intracellular protein to protect against cadmium toxicity. Annu. Rev. Pharmacol. Toxicol..

[4] Wu K.C., Liu J.J., Klaassen C.D. (2012). Nrf2 activation prevents cadmium-induced acute liver injury. Toxicol. Appl. Pharmacol..

[5] Straub P.F., Higham M.L., Tanguy A., Landau B.J., Phoel W.C., Hales L.S., Thwing T.K. (2004). Suppression subtractive hybridization cDNA libraries to identify differentially expressed genes from contrasting fish habitats. Mar. Biotechnol..

[6] Vecchi C., Montosi G., Zhang K., Lamberti I., Duncan S.A., Kaufman R.J., Pietrangelo A. (2009). ER stress controls iron metabolism through induction of hepcidin. Science.

[7] Oliveira S.J., Pinto J.P., Picarote G., Costa V.M., Carvalho F., Rangel M., de Sousa M., de Almeida S.F. (2009). ER stress-inducible factor CHOP affects the expression of hepcidin by modulating C/EBPalpha activity. PLoS One.

[8] Nguyen T., Sherratt P.J., Pickett C.B. (2003). Regulatory mechanisms controlling gene expression mediated by the antioxidant response element. Annu. Rev. Pharmacol. Toxicol..

[9] Rushmore T.H., Morton M.R., Pickett C.B. (1991). The antioxidant responsive element. Activation by oxidative stress and identification of the DNA consensus sequence required for functional activity. J. Biol. Chem..

[10] Tsuji Y., Ayaki H., Whitman S.P., Morrow C.S., Torti S.V., Torti F.M. (2000). Coordinate transcriptional and translational regulation of ferritin in response to oxidative stress. Mol. Cell. Biol..

[11] Braun S., Hanselmann C., Gassmann M.G., auf dem Keller U., Born-Berclaz C., Chan K., Kan Y.W., Werner S. (2002). Nrf2 transcription factor, a novel target of keratinocyte growth factor action which regulates gene expression and inflammation in the healing skin wound. Mol. Cell. Biol..

[12] Wakabayashi N., Shin S., Slocum S.L., Agoston E.S., Wakabayashi J., Kwak M.K., Misra V., Biswal S., Yamamoto M., Kensler T.W. (2010). Regulation of Notch1 signaling by Nrf2: implications for tissue regeneration. Sci. Signal..

[13] Ramos-Gomez M., Kwak M.K., Dolan P.M., Itoh K., Yamamoto M., Talalay P., Kensler T.W. (2001). Sensitivity to carcinogenesis is increased and chemoprotective efficacy of enzyme inducers is lost in nrf2 transcription factor-deficient mice. Proc. Natl. Acad. Sci. USA.

[14] Li J., Stein T.D., Johnson J.A. (2004). Genetic dissection of systemic autoimmune disease in Nrf2-deficient mice. Physiol. Genom..

[15] Itoh K., Wakabayashi N., Katoh Y., Ishii T., Igarashi K., Engel J.D., Yamamoto M. (1999). Keap1 represses nuclear activation of antioxidant responsive elements by Nrf2 through binding to the amino-terminal Neh2 domain. Genes. Dev..

[16] Kobayashi A., Kang M.I., Okawa H., Ohtsuji M., Zenke Y., Chiba T., Igarashi K., Yamamoto M. (2004). Oxidative stress sensor Keap1 functions as an adaptor for Cul3-based E3 ligase to regulate proteasomal degradation of Nrf2. Mol. Cell. Biol..

[17] Rada P., Rojo A.I., Chowdhry S., McMahon M., Hayes J.D., Cuadrado A. (2011). SCF/β-TrCP promotes glycogen synthase kinase 3-dependent degradation of the Nrf2 transcription factor in a Keap1-independent manner. Mol. Cell. Biol..

[18] Li W., Liu H., Zhou J.S., Cao J.F., Zhou X.B., Choi A.M., Chen Z.H., Shen H.H. (2012). Caveolin-1 inhibits expression of antioxidant enzymes through direct interaction with nuclear erythroid 2 p45-related factor-2 (Nrf2). J. Biol. Chem..

[19] Wang H., Liu K., Geng M., Gao P., Wu X., Hai Y., Li Y., Li Y., Luo L., Hayes J.D., Wang X.J., Tang X. (2013). RXRα inhibits the Nrf2-ARE signaling pathway through a direct interaction with the Neh7 domain of Nrf2. Cancer Res..

[20] Gutteridge J.M. (1986). Iron promoters of the Fenton reaction and lipid peroxidation can be released from haemoglobin by peroxides. FEBS Lett..

[21] McCord J.M. (1998). Iron, free radicals, and oxidative injury. Semin. Hematol..

[22] Bayele H.K., Peyssonnaux C., Giatromanolaki A., Arrais-Silva W.W., Mohamed H.S., Collins H., Giorgio S., Koukourakis M., Johnson R.S., Blackwell J.M., Nizet V., Srai S.K. (2007). HIF-1 regulates heritable variation and allele expression phenotypes of the macrophage immune response gene SLC11A1 from a Z-DNA forming microsatellite. Blood.

[23] Torrance J.D., Bothwell T.H. (1968). A simple technique for measuring storage iron concentrations in formalinised liver samples. S. Afr. J. Med..

[24] Ilyin G., Courselaud B., Troadec M.B., Pigeon C., Alizadeh M., Leroyer P., Brissot P., Loréal O. (2003). Comparative analysis of mouse hepcidin 1 and 2 genes: evidence for different patterns of expression and co-inducibility during iron overload. FEBS Lett..

[25] Michaud-Levesque J., Bousquet-Gagnon N., Beliveau R. (2012). Quercetin abrogates IL-6/STAT3 signaling and inhibits glioblastoma cell line growth and migration. Exp. Cell. Res..

[26] Liu J., Li X., Yue Y., Li J., He T., He Y. (2005). The inhibitory effect of quercetin on IL-6 production by LPS-stimulated neutrophils. Cell. Mol. Immunol..

[27] Milenkovic D., Jude B., Morand C. (2013). miRNA as molecular target of polyphenols underlying their biological effects. Free Radic. Biol. Med..

[28] Lesjak M., Hoque R., Balesaria S., Skinner V., Debnam E.S., Srai S.K., Sharp P.A. (2014). Quercetin inhibits intestinal iron absorption and ferroportin transporter expression in vivo and in vitro. PLoS One.

[29] Harada N., Kanayama M., Maruyama A., Yoshida A., Tazumi K., Hosoya T., Mimura J., Toki T., Maher J.M., Yamamoto M., Itoh K. (2011). Nrf2 regulates ferroportin 1-mediated iron efflux and counteracts lipopolysaccharide-induced ferroportin 1 mRNA suppression in macrophages. Arch. Biochem. Biophys..

[30] Liu X.B., Nguyen N.B., Marquess K.D., Yang F., Haile D.J. (2005). Regulation of hepcidin and ferroportin expression by lipopolysaccharide in splenic macrophages. Blood Cells Mol. Dis..

[31] Chiabrando D., Fiorito V., Marro S., Silengo L., Altruda F., Tolosano E. (2013). Cell-specific regulation of Ferroportin transcription following experimentally-induced acute anemia in mice. Blood Cells Mol. Dis..

[32] Nairz M., Theurl I., Ludwiczek S., Theurl M., Mair S.M., Fritsche G., Weiss G. (2007). The co-ordinated regulation of iron homeostasis in murine macrophages limits the availability of iron for intracellular Salmonella typhimurium. Cell. Microbiol..

[33] Dhakshinamoorthy S., Jain A.K., Bloom D.A., Jaiswal A.K. (2005). Bach1 competes with Nrf2 leading to negative regulation of the antioxidant response element (ARE)-mediated NAD(P)H:quinone oxidoreductase 1 gene expression and induction in response to antioxidants. J. Biol. Chem..

[34] Kumar V., Kumar S., Hassan M., Wu H., Thimmulappa R.K., Kumar A., Sharma S.K., Parmar V.S., Biswal S., Malhotra S.V. (2011). Novel chalcone derivatives as potent Nrf2 activators in mice and human lung epithelial cells. J. Med. Chem..

[35] Venugopal R., Jaiswal A.K. (1998). Nrf2 and Nrf1 in association with Jun proteins regulate antioxidant response element-mediated expression and coordinated induction of genes encoding detoxifying enzymes. Oncogene.

[36] Katsuoka F., Motohashi H., Ishii T., Aburatani H., Engel J.D., Yamamoto M. (2005). Genetic evidence that small maf proteins are essential for the activation of antioxidant response element-dependent genes. Mol. Cell. Biol..

[37] Huang M., Qian F., Hu Y., Ang C., Li Z., Wen Z. (2002). Chromatin-remodelling factor BRG1 selectively activates a subset of interferon-α-inducible genes. Nat. Cell. Biol..

[38] Ni Z., Bremner R. (2007). Brahma-related gene 1-dependent STAT3 recruitment at IL-6-inducible genes. J. Immunol..

[39] Zhang J., Ohta T., Maruyama A., Hosoya T., Nishikawa K., Maher J.M., Shibahara S., Itoh K., Yamamoto M. (2006). BRG1 interacts with Nrf2 to selectively mediate HO-1 induction in response to oxidative stress. Mol. Cell. Biol..

[40] Kadam S., Emerson B.M. (2003). Transcriptional specificity of human SWI/SNF BRG1 and BRM chromatin remodeling complexes. Mol. Cell..

[41] Kadam S., McAlpine G.S., Phelan M.L., Kingston R.E., Jones K.A., Emerson B.M. (2000). Functional selectivity of recombinant mammalian SWI/SNF subunits. Genes. Dev..

[42] Rushworth S.A., MacEwan D.J., O'Connell M.A. (2008). Lipopolysaccharide-induced expression of NAD(P)H:quinone oxidoreductase 1 and heme oxygenase-1 protects against excessive inflammatory responses in human monocytes. J. Immunol..

[43] Petrat F., Rauen U., de Groot H. (1999). Determination of the chelatable iron pool of isolated rat hepatocytes by digital fluorescence microscopy using the fluorescent probe, Phen Green SK. Hepatology.

[44] Zhang D.D., Lo S.C., Cross J.V., Templeton D.J., Hannink M. (2004). Keap1 is a redox-regulated substrate adaptor protein for a Cul3-dependent ubiquitin ligase complex. Mol. Cell. Biol..

[45] Kell D.B. (2009). Iron behaving badly: inappropriate iron chelation as a major contributor to the aetiology of vascular and other progressive inflammatory and neurodegenerative diseases. BMC Med. Genom..

[46] Sykiotis G.P., Bohmann D. (2010). Stress-activated cap'n'collar transcription factors in aging and human disease. Sci. Signal.

[47] Kensler T.W., Wakabayashi N., Biswal S. (2007). Cell survival responses to environmental stresses via the Keap1-Nrf2-ARE pathway. Annu. Rev. Pharmacol. Toxicol..

[48] Okada K., Warabi E., Sugimoto H., Horie M., Tokushige K., Ueda T., Harada N., Taguchi K., Hashimoto E., Itoh K., Ishii T., Utsunomiya H., Yamamoto M., Shoda J. (2012). Nrf2 inhibits hepatic iron accumulation and counteracts oxidative stress-induced liver injury in nutritional steatohepatitis. J. Gastroenterol..

[49] Tanaka Y., Aleksunes L.M., Goedken M.J., Chen C., Reisman S.A., Manautou J.E., Klaassen C.D. (2008). Coordinated induction of Nrf2 target genes protects against iron nitrilotriacetate (FeNTA)-induced nephrotoxicity. Toxicol. Appl. Pharmacol..

[50] Silva-Gomes S., Santos A.G., Caldas C., Silva C.M., Neves J.V., Lopes J., Carneiro F., Rodrigues P.N., Duarte T.L. (2014). Transcription factor NRF2 protects mice against dietary iron-induced liver injury by preventing hepatocytic cell death. J. Hepatol..

[51] Yanagawa T., Itoh K., Uwayama J., Shibata Y., Yamaguchi A., Sano T., Ishii T., Yoshida H., Yamamoto M. (2004). Nrf2 deficiency causes tooth decolourization due to iron transport disorder in enamel organ. Genes. Cells.

[52] Thimmulappa R.K., Lee H., Rangasamy T., Reddy S.P., Yamamoto M., Kensler T.W., Biswal S. (2006). Nrf2 is a critical regulator of the innate immune response and survival during experimental sepsis. J. Clin. Invest..

[53] Kwak M.K., Wakabayashi N., Itoh K., Motohashi H., Yamamoto M., Kensler T.W. (2003). Modulation of gene expression by cancer chemopreventive dithiolethiones through the Keap1-Nrf2 pathway. Identification of novel gene clusters for cell survival. J. Biol. Chem..

[54] Herschman H.R. (1991). Primary response genes induced by growth factors and tumor promoters. Annu. Rev. Biochem..

[55] Perron N.R., Brumaghim J.L. (2009). A review of the antioxidant mechanisms of polyphenol compounds related to iron binding. Cell. Biochem. Biophys..

[56] Kim E.Y., Ham S.K., Shigenaga M.K., Han O. (2008). Bioactive dietary polyphenolic compounds reduce nonheme iron transport across human intestinal cell monolayers. J. Nutr..

[57] Kaltwasser J.P., Werner E., Schalk K., Hansen C., Gottschalk R., Seidl C. (1998). Clinical trial on the effect of regular tea drinking on iron accumulation in genetic haemochromatosis. Gut.

[58] Toyokuni S. (1996). Iron-induced carcinogenesis: the role of redox regulation. Free Radic. Biol. Med..

[59] Martines A.M., Masereeuw R., Tjalsma H., Hoenderop J.G., Wetzels J.F., Swinkels D.W. (2013). Iron metabolism in the pathogenesis of iron-induced kidney injury. Nat. Rev. Nephrol..

[60] Kato J., Kobune M., Kohgo Y., Sugawara N., Hisai H., Nakamura T., Sakamaki S., Sawada N., Niitsu Y. (1996). Hepatic iron deprivation prevents spontaneous development of fulminant hepatitis and liver cancer in Long-Evans Cinnamon rats. J. Clin. Invest..

